# De-escalated Teclistamab dosing in relapsed/refractory multiple myeloma: Czech myeloma group real-world evidence analysis

**DOI:** 10.1007/s00277-025-06529-1

**Published:** 2025-08-18

**Authors:** Martin Stork, Jakub Radocha, Jana Mihalyova, Ivan Spicka, Tomas Pika, Alexandra Jungova, Ivanna Boichuk, Klara Mensikova, Jan Straub, Frantisek Sedlak, Jiri Minarik, Petra Krhovska, Denisa Novakova, Michaela Hornakova, Zdenka Knechtova, Nela Sendlerova, Tereza Dekojova, Vladimir Maisnar, Tomas Jelinek, Roman Hajek, Ludek Pour

**Affiliations:** 1 Department of Internal Medicine, Hematology and Oncology, Faculty of Medicine, University Hospital Brno, Masaryk University, Brno, Czech Republic; 2 Department of Internal Medicine – Hematology, Faculty of Medicine in Hradec Kralove, University Hospital Hradec Kralove, Charles University, Hradec Kralove, Czech Republic; 3 Department of Hematooncology, University Hospital Ostrava, Ostrava, Czech Republic; 4 Department of Hematooncology, Faculty of Medicine, University of Ostrava, Ostrava, Czech Republic; 5 st Medical Department, Clinical Department of Hematology of the First Faculty of Medicine, General Teaching Hospital Charles University, Prague, Czech Republic; 6 Department of Hemato-Oncology, Faculty of Medicine and Dentistry, University Hospital Olomouc, Palacky University Olomouc, Olomouc, Czech Republic; 7 Hematology and Oncology Department, Charles University Hospital, Pilsen, Czech Republic

**Keywords:** Multiple myeloma, Teclistamab, Immunotherapy, Real-world-evidence

## Abstract

Teclistamab, a BCMA×CD3 bispecific antibody, demonstrates high efficacy in relapsed/refractory multiple myeloma (RRMM). However, optimal dosing strategies outside clinical trials remain undefined. Thus, we performed a retrospective, multicentre analysis of 73 RRMM patients treated with teclistamab monotherapy at Czech Myeloma Group centres between 2023 and 2025. The study compared efficacy and safety between patients receiving standard weekly dosing and those with reduced-frequency dosing. The whole cohort had a median age of 67 years; 68.5% were penta-refractory. Dosing was de-escalated in 24.7% of patients, typically within one month of treatment initiation. Median progression-free survival (PFS) was 9.41 months and was comparable between weekly and non-weekly groups (9.1 vs. 11.3 months; *p* = 0.141), despite a significantly lower relative dose intensity in the latter (60.5% vs. 87.0%; *p* < 0.001). Infection rates and severe adverse events were similar between groups. A lower incidence of neutropenia was observed with less frequent dosing, but this did not translate into reduced infection burden. In conclusion, in real-world practice, early de-escalation of teclistamab dosing appears to maintain clinical efficacy. These findings support ongoing efforts to individualize treatment schedules with the aim of balancing effectiveness, tolerability, and patient-specific factors in BCMA-targeted therapy.

**Supplementary Information:**

The online version contains supplementary material available at 10.1007/s00277-025-06529-1.

## Introduction

Teclistamab is a bispecific antibody that targets multiple myeloma (MM) by binding to B-cell maturation antigen (BCMA) on MM cells and CD3 on T cells, triggering a T-cell-mediated anti-tumor response. In the MajesTEC-1 trial, it showed high efficacy in heavily pretreated patients with relapsed/refractory MM (RRMM) [1], supported by emerging real-world evidence [2–4]. However, teclistamab carries a high risk of infections due to B-cell depletion and immune dysregulation. Recurrent infections affect ~ 60–75% of patients, mostly related to teclistamab-induced hypogammaglobulinemia [5].

In the MajesTEC-1 trial, patients received an initial step-up dosing of teclistamab followed by a fixed weekly maintenance dose. For those who achieved a complete response after six months of treatment, the dosing frequency could be reduced to biweekly administration [1]. In real-world clinical practice, however, dosing schedules are often adapted for various reasons, potentially deviating from the standardized protocols used in controlled clinical trials.

## Patients and methods

We conducted a retrospective analysis of teclistamab monotherapy in heavily pretreated RRMM patients across all major Czech haematology centres between 2023 and 2025. The primary objective of this study was to compare the efficacy and safety of standard dosing versus reduced-frequency dosing. All data were collected and analysed from the Czech Registry of Monoclonal Gammopathies (RMG) of Czech Myeloma Group (CMG). All participants provided written informed consents approved by institutional Ethics boards in accordance with the latest Helsinki declaration. Data collection cut-off date was 10th January 2025.

The study population consisted of 73 RRMM patients with a median age of 67.0 years (range, 41–83). The median number of prior lines of therapy was 5 (range, 3–13), with 68.5% (50/73) of patients being penta-refractory. Totally 24.7% (18/73) of patients had extramedullary plasmacytomas (EMD, not associated to bone lesions). Of the 59 patients with available cytogenetics, 45.8% (27/59) had two or more high-risk cytogenetic aberrations (HR-CA, defined as t(4;14), t(14;16), del(17p), del(1p32) and gain/amp(1q21)). The median follow-up was 4.9 months (range, 0.3–17.9).

Teclistamab was administered subcutaneously in all patients according to a step-up dosing protocol (0.06 mg/kg on Day 1 and 0.3 mg/kg on Day 4, followed by the first full dose of 1.5 mg/kg on Day 7). Subsequently, in the Weekly group, teclistamab is given at a maintenance dose of 1.5 mg/kg once weekly until disease progression or unacceptable toxicity occurs. In patients who achieved a complete response (CR) or better for at least six months, the dosing frequency of teclistamab was reduced to every other week. Patients who had earlier dosing frequency de-escalation for various reasons were categorized as the Non-weekly group.

Response was assessed according to International Myeloma Working Group criteria. Relative dose intensity (RDI) was counted as the ratio of real cumulative dose (RCD) to maximal ideal dose (MID). Event-free survival (progression free survival - PFS, and overall survival - OS) was defined as the time from treatment initiation to the event or patient’s last follow-up. If the event didn’t occur, the censoring was done at the time of a last follow-up. It was assessed using the Kaplan-Meier methodology and all point estimates include 95% confidence intervals (95% CI). The statistical significance of differences in survival between subgroups was assessed using the log-rank test. The statistical significance of differences in categorical or continuous variables between the subgroups was tested by Fisher Exact test or Mann-Whitney U test. All statistical tests were performed at a significance level of α = 0.05 (all tests two-sided).

## Results

### Response and survival in all patients

The overall response rate (ORR) in evaluable patients was 58.8% (40 out of 68), with 52.9% (36 out of 68) achieving a very good partial response (VGPR) or better. The median PFS was 9.41 months (95% CI: 7.11–not applicable), and the median OS was 15.38 months (95% CI: 10.95–not applicable).

### Prognostic subgroups

Achievement of a deeper response was strongly associated with improved survival outcomes. Patients who achieved a VGPR or better had significantly longer median PFS compared to those with a partial response (PR) or less: 15.38 months (95% CI: 11.18–not estimable) vs. 1.38 months (95% CI: 1.28–3.48), respectively (*p* < 0.001). This benefit was also reflected in estimated 12-month overall survival (OS) rates, which were 75.0% (95% CI: 57.2–98.4) for patients with VGPR or better, compared to 27.8% (95% CI: 12.5–61.8) for those with PR or worse (*p* < 0.001).

Additionally, patients with extramedullary disease (EMD) had shortest median PFS compared to those with para-skeletal plasmacytomas (growing outside of bone lesions) or those with the absence of plasmacytomas: 3.87 months (95% CI: 1.38–not estimable) vs. 10.1 months (95% CI: 6.4– not estimable) vs. not estimable; *p* = 0.059. Moreover, patients harbouring ≥ 2 high-risk HR-CA had shorter median PFS when compared to those with 1 or no HR-CA: 1.84 months (95% CI: 1.38–not estimable) vs. 9.4 months (95% CI: 7.97–not estimable); *p* = 0.022, respectively. A full overview of survival outcomes in whole cohort and subgroups is provided in Supplementary Figs. 1–8.

### Dosing frequency and relative dose intensity

Dosing frequency was modified in 24.7% (18/73) of patients, with 72.2% (13/18) receiving teclistamab every two weeks and 27.8% (5/18) every four weeks. The median time to de-escalation was 1 month (range, 0–3). The most common reason for de-escalation was achievement of a favourable response (55.6%, 10/18), followed by adverse events (33.3%, 6/18) and patient frailty (11.1%, 2/18).

The median relative dose intensity (RDI), calculated as the ratio of actual cumulative dose (RCD) to the maximum ideal dose (MID), was 80.1% (range, 25.3–100.0%) across the entire cohort. Patients in the non-weekly dosing group had a significantly lower median RDI of 60.5% (range, 37.2–93.8%) compared to 87.0% (range, 25.3–100.0%) in the weekly group (*p* < 0.001).

Baseline characteristics were generally balanced between groups. The median age was slightly higher in the non-weekly group (69 years [range, 49–83]) than in the weekly group (66 years [range, 41–85]; *p* = 0.065). Median ECOG performance status was 1 in both groups (range, 0–3; *p* = 0.37). Additional comparisons of baseline characteristics and supportive treatment are provided in Table 1.

**Table 1:** Demographic characteristics and treatment in non-weekly and weekly dosing groups

	Non weekly dosing	Weekly dosing	*p* value‡
Number of pts (%, n/N)	24.7% (18/73)	75.3% (55/73)	-
Median age (y, range)	69.0 (49.0–83.0)	66.0 (41.0–85.0)	0.065
Gender (female, % n/N)	55.6% (10/18)	49.1% (27/55)	0.787
ISS stage (%, 1/2/3)	27.8%/38.9%/33.3%	30.9%/45.5%/23.6%	0.716
ECOG performance status (median, range)	1 (0–3)	1 (0–3)	0.370
No. of previous treatment lines (median, range)	4 (3–9)	5 (3–13)	0.459
Penta-refractory patients	55.6% (10/18)	88.9% (40/55)	0.242
Previous anti-BCMA therapy	5.6% (1/18	14.5% (8/55)	0.436
Extramedullary plasmacytomas	11.1% (2/18)	29.1% (16/55)	0.207
2 or more HR-CA^+^	28.6% (4/14)	48.9% (22/45)	0.227
Without IVIG* administration	27.8% (5/18)	23.6% (13/55)	0.758
Median monthly IVIG* dose (grams/patient)	19.1 (4.0-22.7)	22.4 (4.0-100.0)	0.056
Median follow up (months, range)	5.9 (1.3–13.9)	4.7 (0.3–17.9)	0.327

HR-CA– high-risk cytogenetic aberrations^+^;* intravenous immunoglobulins

**‡** Fisher Exact test or Chi-square test, Mann-Whitney U test

^+^ t(4;14), t(14;16), del(17p), del(1p32) and gain/amp(1q21)

### Survival outcomes by dosing frequency

Progression-free survival (PFS) was comparable between patients receiving weekly and non-weekly teclistamab dosing. The median PFS was 9.1 months (95% CI: 2.7–not estimable) in the weekly group and 11.3 months (95% CI: 6.4–not estimable) in the non-weekly group (*p* = 0.141).

**Fig. 1:**
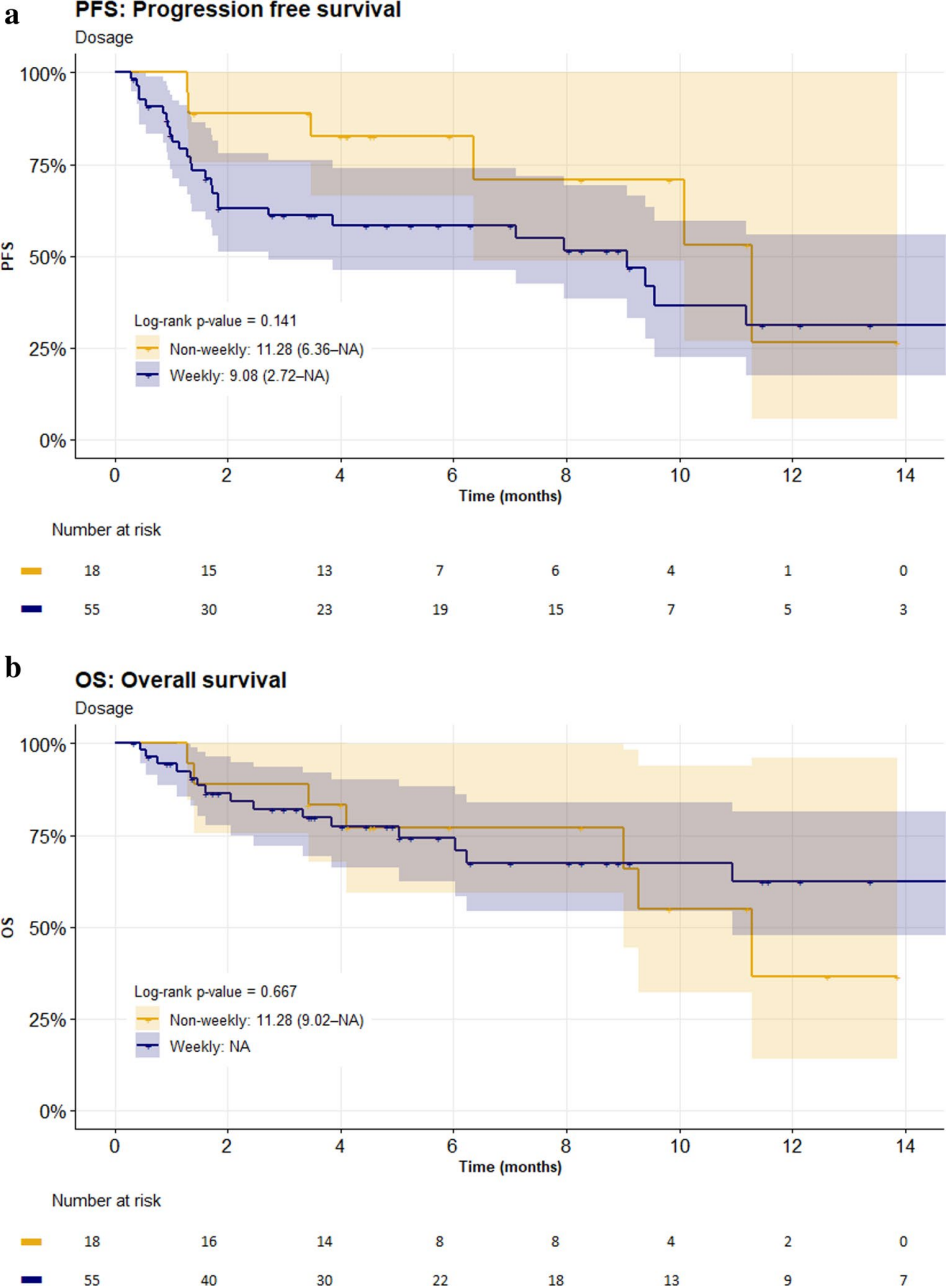
**a** -Progression free survival (PFS) - Non-weekly vs. Weekly dosing**b** - Overall survival (OS) - Non-weekly vs. Weekly dosing

Overall survival (OS) data were still immature at the time of analysis. However, estimated 12-month OS rates suggested a clinically meaningful trend in favour of weekly dosing: 62.3% (95% CI: 47.6–81.5) in the weekly group versus 36.6% (95% CI: 14.0–95.9) in the non-weekly group. This difference did not reach statistical significance (*p* = 0.667). The survival according to dosing frequency is shown in Fig. 1a, b.

### Adverse events by dosing frequency

Cytokine release syndrome (CRS) occurred in 49.3% (36/73) of patients, predominantly grade 1 (39.7%, 29/73), with an additional 9.6% (7/73) experiencing grade 2 events. Immune effector cell-associated neurotoxicity syndrome (ICANS) was reported in two patients (2.7%), both cases being grade 1. Tocilizumab was administered to 9.6% (7/73) of patients to manage CRS and/or ICANS.

Infection rates were comparable between dosing groups when analysed after de-escalation. Infections occurred in 80.0% (44/55) of patients in the weekly group and 66.7% (12/18) in the non-weekly group (*p* = 0.335). Severe infections (grade 3–4) were reported in 29.1% (16/55) of weekly-dosed patients and 50.0% (9/18) of non-weekly patients (*p* = 0.152). One patient in the weekly group died from severe sepsis linked to teclistamab treatment.

Among patients in the non-weekly group, infection incidence remained similar before and after dose de-escalation. A total of 55.6% (10/18) experienced infections prior to de-escalation, and 66.7% (12/18) thereafter (*p* = 0.733). Severe infections occurred in 22.2% (4/18) before de-escalation and in 50.0% (9/18) afterward (*p* = 0.164), indicating a numerical increase that did not reach statistical significance.

When assessing cumulative infection episodes irrespective of timing, the median number of all infection events was 2 (range, 0–12) in the weekly group and 1 (range, 0–11) in the non-weekly group (*p* = 0.730). However, the median number of grade 3–4 infection episodes was significantly higher in the non-weekly group (median 1; range, 0–3) compared to the weekly group (median 0; range, 0–4; *p* = 0.033).

Neutropenia occurred more frequently in patients receiving weekly dosing: 61.8% (34/55) versus 22.2% (4/18) in the non-weekly group (*p* = 0.006). The incidence of severe neutropenia (grade 3–4) was similar between groups (23.6% vs. 16.7%; *p* = 0.745). In the non-weekly group, severe neutropenia occurred in 5.5% (1/18) of patients prior to de-escalation and in 16.7% (3/18) after de-escalation (*p* = 0.603). Rates of anemia and thrombocytopenia were comparable between the two groups. Detailed toxicity data are presented in Table 2. Severe toxicity before and after de-escalation is summarized in the Supplementary Table 1.

**Table 2:** Toxicity according to dosing scheme

Adverse event	Gr.	Non-weekly dosing	Weekly dosing	*p* value*
CRS	Gr.1–2	44.4% (8/18)	49.1% (27/55)	0.790
ICANS	Gr.1	11.1% (2/18)	0.0%	1.000
Infection^+^	Total	66.7% (12/18)	80.0% (44/55)	0.335
	Gr.3–4	50.0% (9/18)	29.1% (16/55)	0.152
	Gr.5	0.0%	1.8% (1/55)	1.000
Anemia^+^	Total	38.9% (7/18)	63.6% (35/55)	0.099
	Gr.3–4	5.6% (1/18)	7.3% (4/55)	1.000
Thrombopenia^+^	Total	22.2% (4/18)	41.8% (23/55)	0.167
	Gr.3–4	16.7% (3/18)	10.9% (6/55)	0.680
Neutropenia^+^	Total	22.2% (4/18)	61.8% (34/55)	**0.006**
	Gr.3–4	16.7% (3/18)	23.6% (13/55)	0.745

CRS – Cytokine Release Syndrome; ICANS – Immune effector Cell Associated Neurotoxicity Syndrome; * Fisher exact test; ^+^ - counted in the non-weekly group after the de-escalation;

**Bold values** are statistically significant

##  Discussion

This multicentre real-world study demonstrates that teclistamab dosing in routine clinical practice often diverges from the fixed weekly maintenance schedule employed in the MajesTEC-1 trial. Despite less frequent dosing, PFS remained comparable to weekly dosing, indicating sustained efficiency in responding patients.

Clinical data on dosing de-escalation of anti-BCMA bispecific antibodies remain limited. In a real-world study, 32% (25/77) of patients transitioned to every-other-week dosing of teclistamab after three months due to toxicity or achieving partial response. At six months, the progression-free survival rate was 94% among these patients, compared to 52% in the overall cohort, likely reflecting positive selection of responders [6]. For elranatamab, the MagnetisMM-3 trial showed that responders could reduce dosing from weekly to every-other-week after six months, then to every-four-week after another six months without losing efficacy [7]. Our data uniquely show the median time to dosing frequency de-escalation was just one month—much shorter than previous studies. Current clinical trials are designed to evaluate both the efficacy and safety of teclistamab at reduced dosing as a monotherapy [8], as well as in combination with other antimyeloma agents [9, 10].

Despite maintained efficacy in non-weekly dosing, no reduction of infection events was observed. This may be due to the prolonged impact of teclistamab on immunoglobulin production, extending beyond the 2- or 4-week off-therapy periods. Variability in intravenous immunoglobulin administration and supportive care across real-world settings also likely contributes to generally inconsistent toxicity outcomes [5, 11, 12]. In our country, immunoglobulin substitution practices vary between centers, with a significant shift from secondary to primary prophylaxis during the study duration. Based on these findings, primary prophylaxis with intravenous immunoglobulins may play an important role in infection management during anti-BCMA therapy, potentially more so than dose reductions. Moreover, response to treatment was the primary reason for dose de-escalation, but frailty and recurring infections also contributed, potentially biasing adverse event reports in the non-weekly group. Also, these patients were generally older. While bispecific antibodies’ efficacy is unaffected by age [1, 6, 13], elderly patients are more susceptible to infections and hematologic toxicity. Both groups were balanced regarding EMD and high-risk cytogenetics, which negatively influenced prognosis both in our study and in so far published works [1, 3].

We noted a reduced incidence of neutropenia with the non-weekly dosing schedule. Since BCMA is not typically expressed on myeloid cells or neutrophils, this might result from less immune cell-mediated myelosuppression due to lower teclistamab plasma levels [13]. The impact of teclistamab dosing frequency on myeloid cells remains unclear and warrants further study.

The OS data, though still immature, seems to favor weekly dosing. Factors such as patient age or alternative treatments like GPRC5D or FCRH5 bispecific antibodies for teclistamab-refractory patients may influence results [14, 15]. More research is needed on how reduced anti-BCMA bispecific antibody dosing affects subsequent therapy efficacy, particularly regarding T-cell exhaustion [16, 17].

The retrospective nature of our study, small sample size and the variability in clinical decision-making processes are limitations that should be acknowledged. However, our findings offer valuable insights into the real-world application of teclistamab and may guide future research efforts aimed at optimizing dosing protocols to balance efficacy, safety, and treatment costs. Our approach may also assist clinicians with teclistamab dosing decisions in challenging cases.

Taken together, in a real-world setting, early de-escalation of teclistamab dosing did not compromise efficacy especially in responding patients. However, reduced dosing was not associated with a lower rate of infectious complications. These findings suggest that flexible, response-adapted dosing strategies may be appropriate in select patients, particularly those with frailty or tolerability concerns. Further prospective studies are needed to validate these observations and to better define the impact of dosing modifications on long-term outcomes, immune recovery, and subsequent therapy sequencing.

## Supplementary Information

Below is the link to the electronic supplementary material.

Supplementary file 1 (DOCX 125KB)

## Data Availability

No datasets were generated or analysed during the current study.
