# Supplementary material for: De-escalated Teclistamab dosing in relapsed/refractory multiple myeloma: Czech myeloma group real-world evidence analysis
Source: Ann Hematol. 2025 Aug 18;104(8):4141–7. doi: 10.1007/s00277-025-06529-1 (PMC12432020; doi:10.1007/s00277-025-06529-1)
Supplement: Supplementary file 1 — (DOCX 125KB) [file 277_2025_6529_MOESM1_ESM.docx]

**Supplementary materials**

**Supplementary figure 1** Progression free survival (PFS) – all patients.


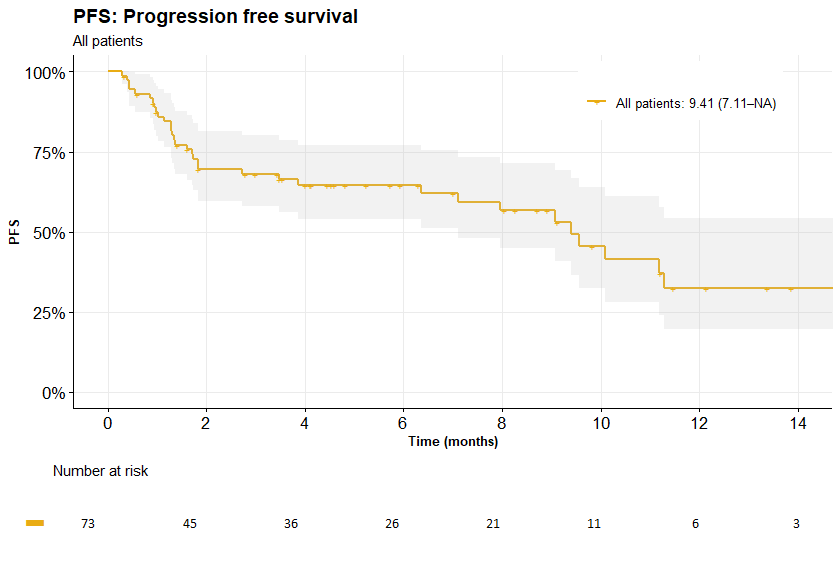


| **PFS** |  |  |
| --- | --- | --- |
|  |  |  |
| n valid | 73 |  |
| n events (%) | 34 (46.6%) |  |
| KM median (95% CI) | 9.4 (7.1–NA) |  |
| **Probability at time, % (95% CI):** | ⠀ |  |
| 2 months | 69.7 (59.6–81.4) |  |
| 4 months | 64.6 (54.1–77.2) |  |
| 6 months | 64.6 (54.1–77.2) |  |
| 8 months | 56.6 (44.9–71.4) |  |
| 10 months | 45.5 (32.4–63.9) |  |
| 12 months | 32.6 (19.5–54.3) |  |

**Supplementary figure 2** Overall survival (OS) – all patients.


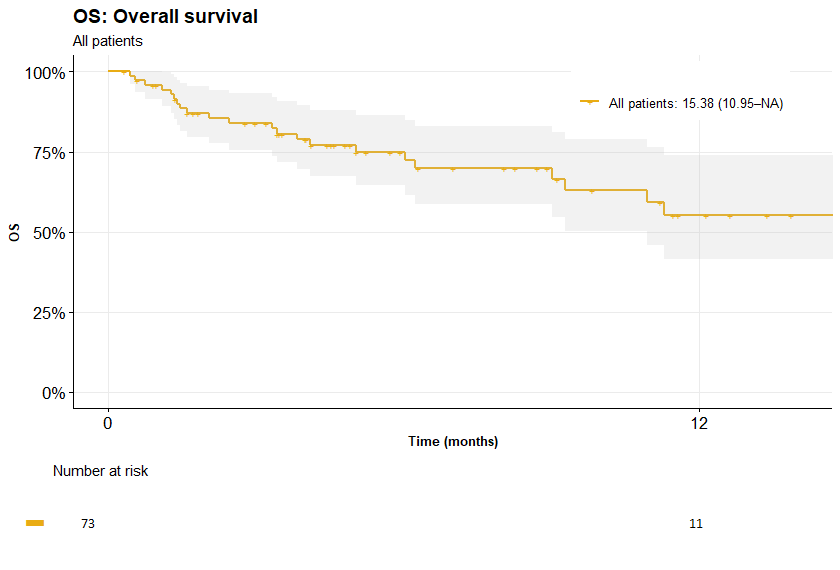


| **OS** |  |  |
| --- | --- | --- |
|  |  |  |
| n valid | 73 |  |
| n events (%) | 23 (31.5%) |  |
| KM median (95% CI) | 15.4 (11.0–NA) |  |
| **Probability at time, % (95% CI):** | ⠀ |  |
| 2 months | 87.0 (79.5–95.3) |  |
| 4 months | 78.8 (69.5–89.4) |  |
| 6 months | 74.8 (64.6–86.5) |  |
| 8 months | 69.8 (58.6–83.1) |  |
| 10 months | 63.0 (50.3–78.9) |  |
| 12 months | 55.3 (41.4–73.8) |  |

**Supplementary figure 3** Progression free survival (PFS) – best response.


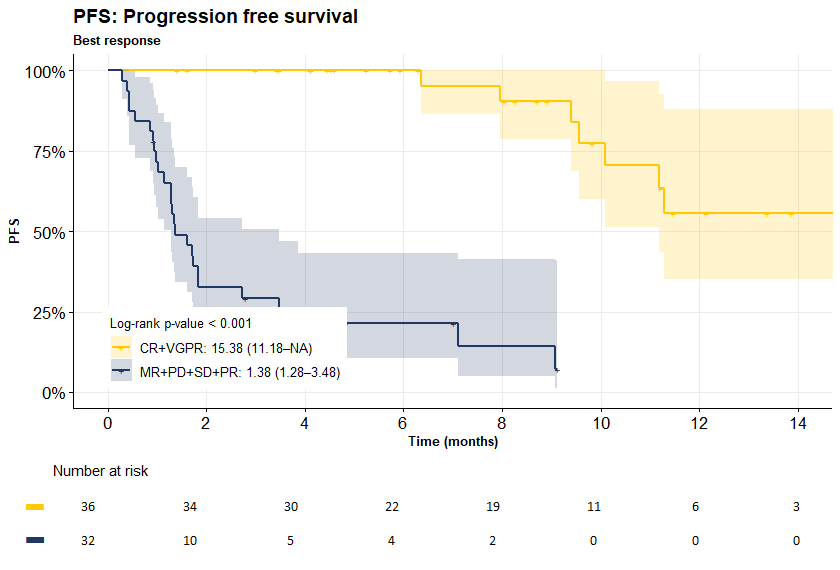


| **PFS** | **CR+VGPR** | **MR+PD+SD+PR** | **p-value of** |
| --- | --- | --- | --- |
|  |  |  | **log-rank test** |
| n valid | 36 | 32 | **<0.001** |
| n events (%) | 8 (22.2%) | 26 (81.2%) |  |
| KM median (95% CI) | 15.4 (11.2–NA) | 1.4 (1.3–3.5) |  |
| **Probability at time, % (95% CI):** | ⠀ | ⠀ |  |
| 2 months | 100.0 (100.0–100.0) | 32.6 (19.6–54.1) |  |
| 4 months | 100.0 (100.0–100.0) | 21.4 (10.6–43.2) |  |
| 6 months | 100.0 (100.0–100.0) | 21.4 (10.6–43.2) |  |
| 8 months | 90.5 (78.8–100.0) | 14.2 (4.9–41.4) |  |
| 10 months | 77.6 (60.1–100.0) | – |  |
| 12 months | 55.5 (35.1–87.9) | – |  |

**Supplementary figure 4** Overall survival (OS) – best response.


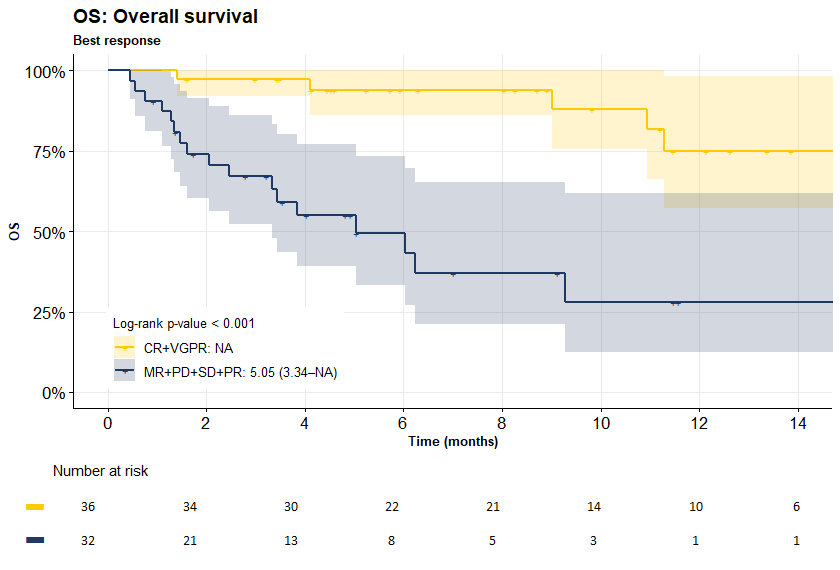


| **OS** | **CR+VGPR** | **MR+PD+SD+PR** | **p-value of** |
| --- | --- | --- | --- |
|  |  |  | **log-rank test** |
| n valid | 36 | 32 | **<0.001** |
| n events (%) | 6 (16.7%) | 17 (53.1%) |  |
| KM median (95% CI) | – | 5.0 (3.3–NA) |  |
| **Probability at time, % (95% CI):** | ⠀ | ⠀ |  |
| 2 months | 97.2 (92.0–100.0) | 74.2 (60.2–91.4) |  |
| 4 months | 97.2 (92.0–100.0) | 55.0 (39.2–77.1) |  |
| 6 months | 94.0 (86.2–100.0) | 49.5 (33.3–73.5) |  |
| 8 months | 94.0 (86.2–100.0) | 37.1 (21.1–65.2) |  |
| 10 months | 88.1 (75.6–100.0) | 27.8 (12.5–61.8) |  |
| 12 months | 75.0 (57.2–98.4) | 27.8 (12.5–61.8) |  |

**Supplementary Figure 5** Progression free survival (PFS) – Extraosseous plasmacytomas in multiple myeloma (EMM) – Extramedullary (EMD) plasmacytomas vs. Para-skeletal (PS) plasmacytoma vs. No plasmacytomas


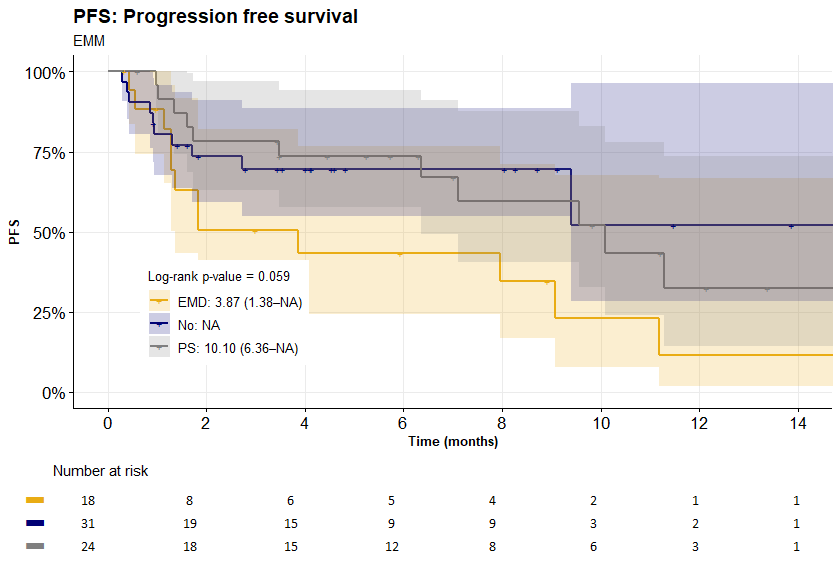


| **PFS** | **EMD** | **No** | **PS** | **p-value of** |
| --- | --- | --- | --- | --- |
|  |  |  |  | **log-rank test** |
| n valid | 18 | 31 | 24 | 0.059 |
| n events (%) | 13 (72.2%) | 10 (32.3%) | 11 (45.8%) |  |
| KM median (95% CI) | 3.9 (1.4–NA) | – | 10.1 (6.4–NA) |  |
| **Probability at time, % (95% CI):** | ⠀ | ⠀ | ⠀ |  |
| 2 months | 50.4 (31.0–81.9) | 73.5 (59.3–91.1) | 78.3 (63.1–97.1) |  |
| 4 months | 43.2 (24.4–76.6) | 69.6 (54.8–88.5) | 73.7 (57.6–94.2) |  |
| 6 months | 43.2 (24.4–76.6) | 69.6 (54.8–88.5) | 73.7 (57.6–94.2) |  |
| 8 months | 34.6 (16.8–71.1) | 69.6 (54.8–88.5) | 59.5 (40.5–87.5) |  |
| 10 months | 23.0 (7.9–67.7) | 52.2 (28.2–96.5) | 52.1 (32.7–83.0) |  |
| 12 months | 11.5 (2.0–66.7) | 52.2 (28.2–96.5) | 32.6 (14.4–73.6) |  |

**Supplementary Figure 6** Overall survival (OS) – Extraosseous plasmacytomas in multiple myeloma (EMM) – Extramedullary (EMD) plasmacytomas vs. Para-skeletal (PS) plasmacytoma vs. No plasmacytomas


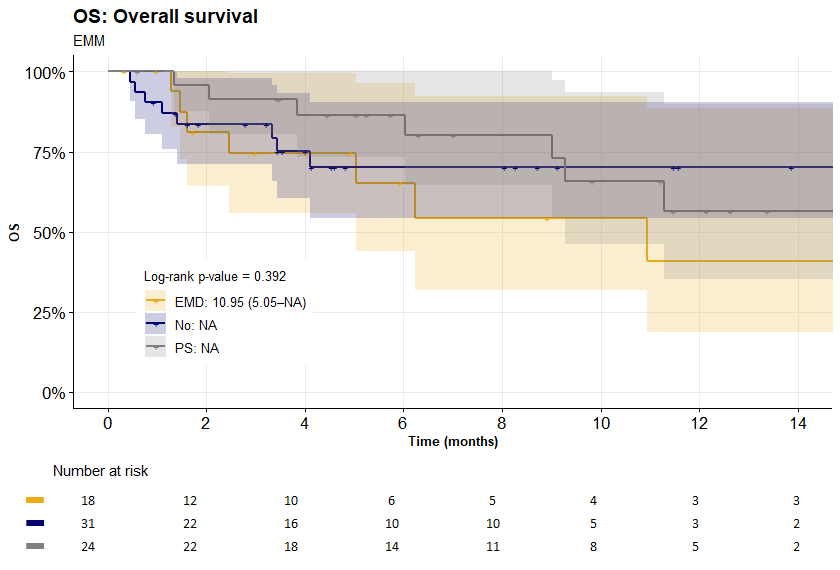


| **OS** | **EMD** | **No** | **PS** | **p-value of** |
| --- | --- | --- | --- | --- |
|  |  |  |  | **log-rank test** |
| n valid | 18 | 31 | 24 | 0.392 |
| n events (%) | 8 (44.4%) | 8 (25.8%) | 7 (29.2%) |  |
| KM median (95% CI) | 11.0 (5.0–NA) | – | – |  |
| **Probability at time, % (95% CI):** | ⠀ | ⠀ | ⠀ |  |
| 2 months | 81.2 (64.2–100.0) | 83.5 (71.3–97.8) | 95.7 (87.7–100.0) |  |
| 4 months | 74.5 (55.7–99.6) | 75.1 (60.6–93.2) | 86.5 (73.4–100.0) |  |
| 6 months | 65.2 (44.1–96.4) | 70.1 (54.4–90.5) | 86.5 (73.4–100.0) |  |
| 8 months | 54.3 (32.0–92.3) | 70.1 (54.4–90.5) | 80.3 (64.5–100.0) |  |
| 10 months | 54.3 (32.0–92.3) | 70.1 (54.4–90.5) | 65.7 (46.1–93.7) |  |
| 12 months | 40.7 (18.8–88.4) | 70.1 (54.4–90.5) | 56.3 (35.3–89.8) |  |

**Supplementary Figure 7** Progression free survival (PFS) – Two or more high-risk cytogenetic aberration* (“double hit multiple myeloma”)


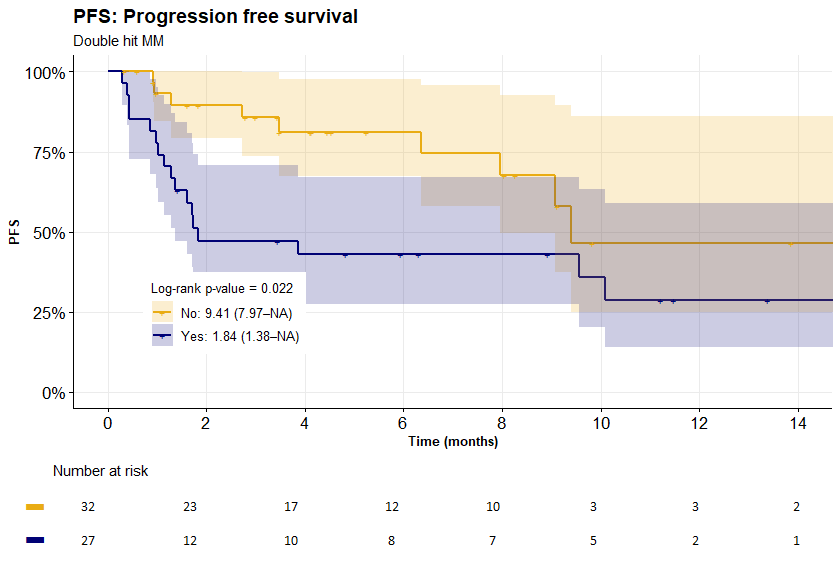


| **PFS** | **No** | **Yes** | **p-value of** |
| --- | --- | --- | --- |
|  |  |  | **log-rank test** |
| n valid | 32 | 27 | **0.022** |
| n events (%) | 10 (31.2%) | 17 (63.0%) |  |
| KM median (95% CI) | 9.4 (8.0–NA) | 1.8 (1.4–NA) |  |
| **Probability at time, % (95% CI):** | ⠀ | ⠀ |  |
| 2 months | 89.6 (79.2–100.0) | 47.2 (31.5–70.8) |  |
| 4 months | 81.2 (67.5–97.7) | 42.9 (27.5–67.0) |  |
| 6 months | 81.2 (67.5–97.7) | 42.9 (27.5–67.0) |  |
| 8 months | 67.7 (49.5–92.6) | 42.9 (27.5–67.0) |  |
| 10 months | 46.4 (25.0–86.1) | 35.8 (20.2–63.4) |  |
| 12 months | 46.4 (25.0–86.1) | 28.6 (13.9–58.8) |  |

*** t(4;14), t(14;16), del(17p), del(1p32) and gain/amp(1q21)

**Supplementary Figure 8** Overall survival (OS) – Two or more high-risk cytogenetic aberration* (“double hit multiple myeloma”)


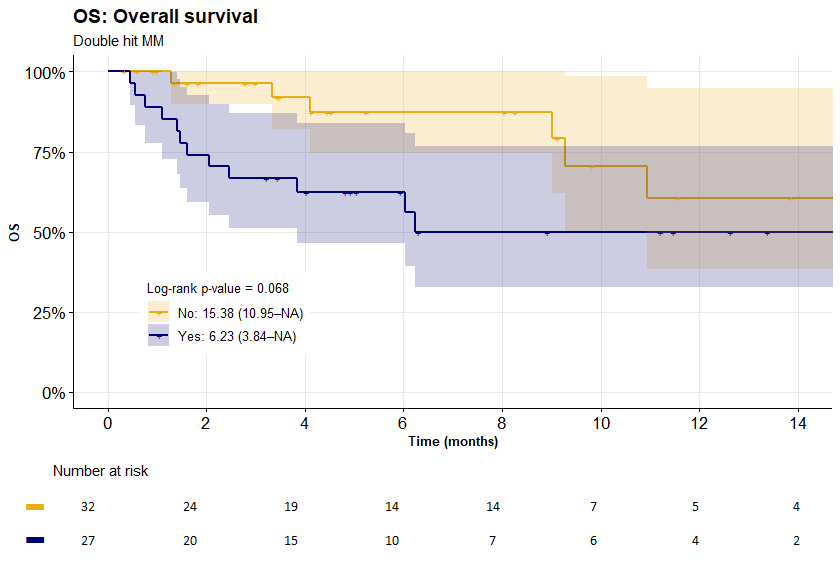


| **OS** | **No** | **Yes** | **p-value of** |
| --- | --- | --- | --- |
|  |  |  | **log-rank test** |
| n valid | 32 | 27 | 0.068 |
| n events (%) | 7 (21.9%) | 12 (44.4%) |  |
| KM median (95% CI) | 15.4 (11.0–NA) | 6.2 (3.8–NA) |  |
| **Probability at time, % (95% CI):** | ⠀ | ⠀ |  |
| 2 months | 96.4 (89.8–100.0) | 74.1 (59.3–92.6) |  |
| 4 months | 92.0 (82.0–100.0) | 62.5 (46.5–84.0) |  |
| 6 months | 87.2 (74.5–100.0) | 62.5 (46.5–84.0) |  |
| 8 months | 87.2 (74.5–100.0) | 50.0 (32.6–76.7) |  |
| 10 months | 70.5 (50.4–98.6) | 50.0 (32.6–76.7) |  |
| 12 months | 60.4 (38.4–94.9) | 50.0 (32.6–76.7) |  |

*** t(4;14), t(14;16), del(17p), del(1p32) and gain/amp(1q21)

**Supplementary table 1** – Severe adverse events before and after de-escalation

| Adverse event | Before de-escalation | After de-escalation | p-value* |
| --- | --- | --- | --- |
| Infections gr.3-4 | 22.4 % (4/18) | 50.0 % (9/18) | 0.164 |
| Neutropenia gr. 3-4 | 5.5 % (1/18) | 16.7 % (3/18) | 0.603 |
| Thrombopenia gr.3-4 | 5.5 % (1/18) | 16.7 % (3/18) | 0.603 |
| Anemia gr.3-4 | 5.5 % (1/18) | 5.5 % (1/18) | 1.000 |

*Fisher exact test
